# Efficacy and safety of dihydroartemisinin–piperaquine for the treatment of uncomplicated *Plasmodium falciparum* and *Plasmodium vivax* malaria in Papua and Sumatra, Indonesia

**DOI:** 10.1186/s12936-022-04101-0

**Published:** 2022-03-19

**Authors:** Puji B. S. Asih, Ismail E. Rozi, Farahana K. Dewayanti, Suradi Wangsamuda, Syarifah Zulfah, Marthen Robaha, Jonny Hutahaean, Nancy D. Anggraeni, Marti Kusumaningsih, Pranti S. Mulyani, Elvieda Sariwati, Herdiana H. Basri, Maria Dorina G. Bustos, Din Syafruddin

**Affiliations:** 1 Malaria and Vector Resistance Unit, Eijkman Institute for Molecular Biology, Jakarta, Indonesia; 2 Jambi Provincial Health Department, Jambi City, Jambi Indonesia; 3 Papua Provincial Health Department, Jayapura City, Papua Indonesia; 4 Malaria Sub-Directorate, Ministry of Health, Jakarta, Republic of Indonesia; 5 World Health Organization, Country Office for Indonesia, Jakarta, Indonesia; 6 World Health Organization, Country Office for Thailand, Nonthaburi, Thailand; 7 Department of Parasitology, Faculty of Medicine, University of Hasanuddin, Makasar, Indonesia

**Keywords:** Therapeutic efficacy, Hihydroartemisinin–piperaquine (DHA–PPQ), *P. falciparum*, *P. vivax*, Indonesia

## Abstract

**Background:**

Dihydroartemisinin–piperaquine (DHA–PPQ) has been adopted as first-line therapy for uncomplicated falciparum malaria in Indonesia since 2010. The efficacy of DHA–PPQ was evaluated in 2 sentinel sites in Keerom District, Papua and Merangin District, Jambi, Sumatra from April 2017 to April 2018.

**Methods:**

Clinical and parasitological parameters were monitored over a 42-day period following the World Health Organization standard in vivo protocol and subjects meeting the inclusion criteria were treated with DHA–PPQ once daily for 3 days, administered orally.

**Results:**

In Papua, 6339 subjects were screened through active and passive cases detection. Of the 114 falciparum and 81 vivax cases enrolled, 102 falciparum and 80 vivax cases completed the 42 day follow up, and 12 falciparum and 1 vivax cases were either lost to follow up or withdrawn. Kaplan–Meier analysis of microscopy readings of 102 falciparum cases revealed 93.1% (95% CI 86.4–97.2) as Adequate Clinical and Parasitological Response (ACPR). No delay in parasite clearance nor severe adverse reaction was observed. Recurrent parasites of *Plasmodium falciparum* were detected in 7 cases and categorized as late treatment failures (LTF) at days 21, 35, and 42 and one of which was reinfected by *Plasmodium vivax* at day 42. Two cases were confirmed as recrudescent infection and 4 were re-infection. The PCR-corrected DHA–PPQ efficacy for *P. falciparum* was 97.9% (95% CI 92.7–99.7). Of the 80 cases of *P. vivax* that were followed up, 71 cases were completely cured and classified as ACPR (88.8%, 95% CI 79.7–94.7) and 9 cases showed recurrent infection at days 35 and 42, and classified as LTF. In Sumatra, of the 751 subjects screened, 35 vivax subjects enrolled, 34 completed the 42 day follow up. Thirty-three cases were completely cured and classified as ACPR (97.1%, 95% CI 84.7–99.9) and 1 recurrent infection was observed and classified as LTF. No delay in parasite clearance nor severe adverse reaction was observed. Analysis of the *Pfk13* gene in *P. falciparum* cases from Papua revealed no mutations associated with artemisinin resistance in the 20 SNPs previously reported. Analysis of the *Pfpm2* gene at day 0 and day of recurrence in recrudescent cases revealed the same single copy number, whereas 3 of the 4 re-infection cases carried 2–3 *Pfpm2* gene copy numbers.

**Conclusion:**

Treatment of falciparum and vivax malaria cases with DHA–PPQ showed a high efficacy and safety.

## Background

In Indonesia, reports to date revealed that artemisinin-based combination therapy (ACT), particularly dihydroartemisinin–piperaquine (DHA–PPQ), is highly effective to treat any human malaria cases. Although certain studies reported few cases of delayed parasite clearance [1], this evidence was not linked to the artemisinin resistance. Subsequent analysis on the cases revealed that the delay may be related to the higher parasite load as the parasite is eventually eliminated by day 7. Therefore, routine monitoring of the therapeutic efficacy of ACT is essential for making timely changes of treatment policy. It can also help to detect early changes in the parasite susceptibility to anti-malarial drugs [2–4].

Malaria control programme in Indonesia has successfully brought down the malaria cases within the last few decades and in 2017, more than half of the district and municipality have been certified as malaria free areas. However, malaria cases remain high in eastern provinces, such as Papua, West Papua, Molucca and East Nusa Tenggara. In Western part of the country, malaria is either eliminated or significantly reduced and only several malaria foci left in Sumatra, Java, Bali and Kalimantan. In 2020, Indonesia reported 254,055 malaria cases with Annual Parasite Incidence (API) 0.94 cases per 1000 population and 74% of infections reported from Papua Province [5]. The malaria problem in Indonesia represents a unique archipelago setting that is entirely different with that of Africa. The malaria control programme relies on three pillars, including early diagnosis and prompt treatment, provision of long-lasting insecticidal nets (LLIN) and indoor residual spraying (IRS) [6]. Unfortunately, the health care facilities in remote setting where malaria is highly endemic does not always meet the requirement to provide necessary service to the people. The absence of microscopists and vector control officers reduce the effectiveness of the pillar and also provision of diagnosis and prompt treatment. To avoid the unnecessary anti-malarial drug deployment, the Ministry of Health has set a treatment guideline in which anti-malarial drugs will only be given to laboratory confirmed cases, either by microscopy or rapid diagnostic test (RDT). In Indonesia, follow up of the malaria treatment is rarely done and, therefore, supervisory treatment has been recommended to ensure that the persons indeed consumed the anti-malarial as prescribed. The objective of this study is to assess the therapeutic efficacy and safety of DHA–PPQ for the treatment of uncomplicated *Plasmodium falciparum* and *Plasmodium vivax* malaria in Indonesia. The additional objective is to observe the gametocyte carriage during the follow-up period.

## Methods

### Study site

**Fig. 1:**
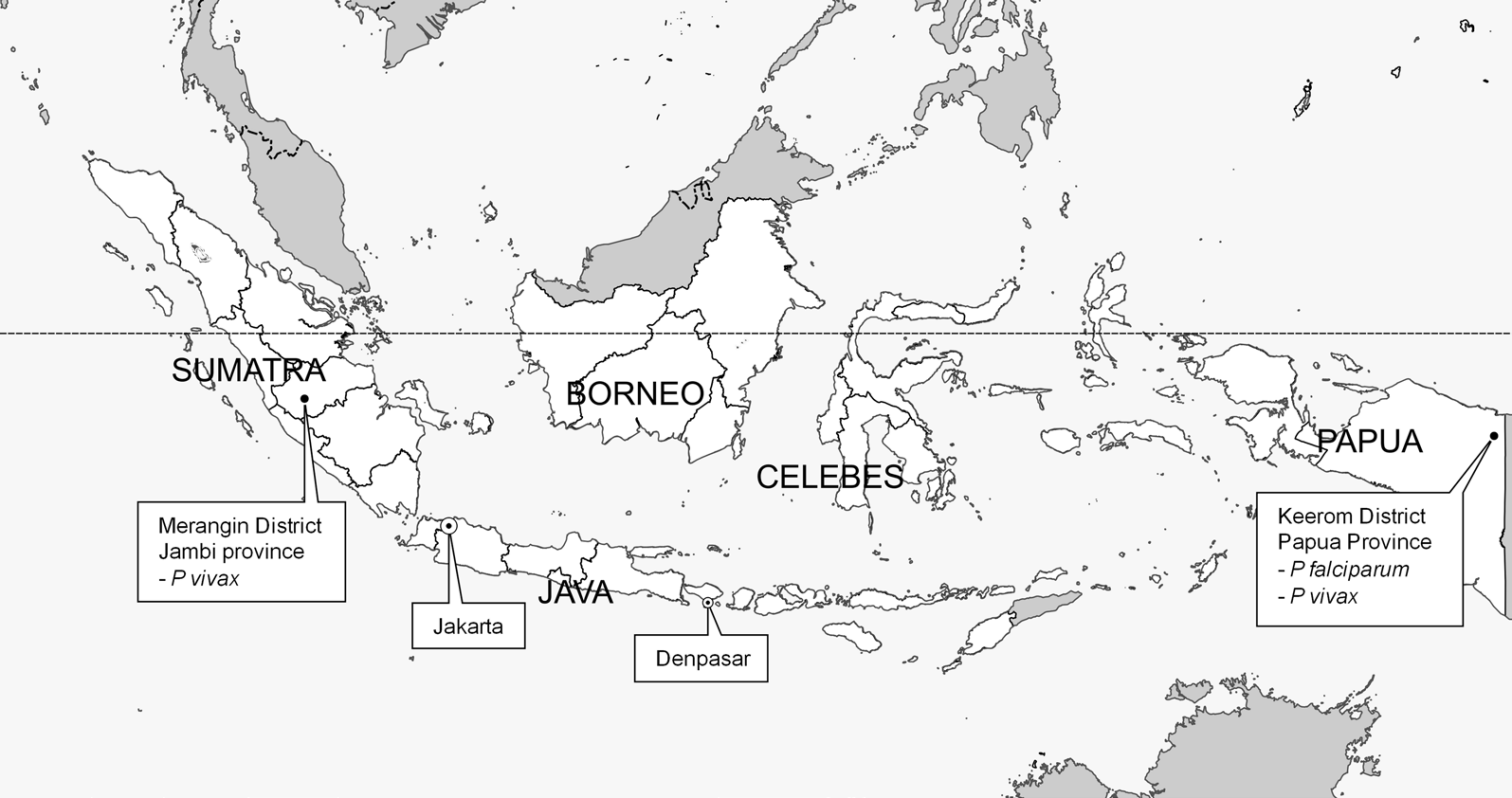
Study Sites in Papua and Sumatra Islands. Map from Natural Earth. https://www.naturalearthdata.com/

The study was conducted in Keerom District, Papua and Merangin District, Jambi, Sumatra from April 2017–April 2018 (Fig. 1). The population ranged from about ~ 55.799 people in Keerom District, Papua and about ~ 377.905 people in Merangin District, Jambi, Sumatra in 2017 [7]. The selected sentinel sites were based on the fact that both sites had high annual parasite incidence (API). A total API in Papua in 2015 until 2018 was 31,93; 45,85; 59; and 52.99 cases per 1000 population respectively. In the meantime, in Jambi Sumatra API in 2015–2018 showed 0.47; 0.14; 0.04; and 0.06 [8].

### In vivo drug efficacy study

The study was designed as one arm prospective evaluation of clinical and parasitological responses to directly observed treatment for uncomplicated malaria. Until 2017, as the treatment failure rate to DHP–PPQ in Papua and Sumatra were unknown, a rate of 30% had been chosen. At a confidence level of 95% and a precision around the estimate of 10%, and with a 20% increased to allowed lost to follow-up and withdrawn during the 42-day follow-up period, it was estimated a total of 120 patients (60 with *P. falciparum* and 60 with *P. vivax* malaria) recruited. Participants were recruited from malaria-infected persons found during active case detection (ACD) or passive case detection (PCD) in Papua and Sumatra. Persons were aged between 1 and 65 years, weighed more than 5 kg, had fever or history of fever in the preceding 24 h, with slide-confirmed malaria with parasitaemia of ≥ 500/ul asexual parasites for *P. falciparum* and ≥ 250/ul asexual parasites for *P. vivax.* Persons were excluded with the following exclusion criteria: pregnant, had a history of allergy to the study drugs or study drug’s derivative, had previously completed treatment with an anti-malarial drug in the preceding 2 weeks, or had a medical history of untreated hypertension or chronic heart, kidney, or liver disease [9].

### Laboratory procedures

Finger prick was performed to obtain blood to prepare thick and thin blood smears haemoglobin measurements on day 0 with blood volume > 5 g/dL, and for dried blood spots (DBS) on filter paper using 3MM Whatman (GE Healthcare, Buckinghamshire,

UK) and kept in individual plastic zip lock for parasite genotyping. A standard physical examination, blood smears and DBS were also collected from finger pricks on days 1, 2, 3, 7, 14, 21, 28, 35 and 42 [9] with time windows 1 day before/after for days 7, 14, 21, 28, 35 and 42. Smears were read by expert microscopists. All vivax study participants were checked for G6PD deficiency using CareStart™ G6PD deficiency screening test and followed the procedures from the manufacturer. A Giemsa 3% dilution was prepared and used to stain the blood smears for 60 min. Parasite density was determined with parasite count per 200 white blood cells in thick smears. A blood slide was considered negative when examination of 1000 white blood cells or 100 fields containing at least 10 white blood cells per field reveals no asexual parasites. Two microscopists read all the slides independently and counted the parasite density. The differences between the two microscopists in species diagnosis and in parasite density of > 50% were re-examined by a third independent microscopist, and parasite density were counted by averaging the two closest counts.

### Anti-malarial therapy

All study participants were given a treatment of dihydroartemisinin (DHA) and piperaquine (PPQ) from primary health centre (DHP-Primal, manufactured by KBN-Zheijang Pharmaceutical Co., Ltd; under license of Beijing Holley-Cotec Pharmaceticals Co., Ltd), containing 40 mg DHA and 320 mg PPQ per tablet and was administered once a day for 3 days in front of study team, as a weight per dose regimen of 2.25 and 18 mg/kg of DHA–PPQ [10] and followed-up weekly for 42 days. The DHA–PPQ was given after taking biscuits, milk or bread provided by this study. The participants were observed for 30 min after DHA–PPQ administration for adverse reactions or vomiting. Any participants who vomit during this observation period were re-treated with the same dose and if vomit again the participants were drawn and given rescue therapy. Parasitological responses and classification of new infection, including lost to follow up and protocol violation were classified according to criteria of the World Health Organization (WHO) [2, 9]. Adverse events such as nausea, vomiting, headache observed during the study were recorded. Primaquine therapy was not provided during the follow up days or until discontinuation from the study i.e., day of recurrence or day 42 for falciparum and vivax cases. The dose of primaquine given was 0.25 mg/kg BW daily for a 14-days for vivax cases and single dose for falciparum cases, administered according to guidelines from Ministry of Health of Indonesia [10]. Primaquine was given only for study participants who indicated normal range in G6PD deficiency test and age > 1 y.o. If participants met the criteria for therapeutic failure, participants were given second line malaria treatment according to current national programme [10].

### Genotyping of *Plasmodium falciparum*

Genomic DNA (on day of enrolment and day of recrudescence) was extracted from DBS using Chelex-100 ion exchanger (Bio-Rad Laboratories, Hercules, CA) according to a previously published procedure [11]. Extracted DNA was either used immediately for Polymerase Chain Reaction (PCR) assays or stored at − 20 °C for later analysis. Genotyping using the genes for merozoite surface protein 1(MSP1), MSP2, and glutamate-rich protein (GLURP) was performed in certain participants to distinguish between pre-treatment and recrudescent parasites [12]. The amplicons from 3 genes above were visualized in agarose gel. The differences length of the amplicons between day 0 and day recrudescence among the 3 genes above were calculated as new infection and override the results of genotyping any other marker.

### Amplification of *Pfk13* gene

Amplification of *Pfk13* gene for artemisinin resistance was performed according to previously published method. The DNA was amplified by nested PCR and sanger sequencing method to detect the SNPs: G449A, N458Y, T474I; M476I; A481V; Y493H; T508N; P527T; G533S; N537I; R539T; I543T; P553L; R561H; V568G; P574L; C580Y; D584V; E612D; S623C of *P. falciparum* K13 [13–19]. BioEdit alignment editor was used to detect the SNPs in DNA sample and reference sequence.

### Quantitative PCR to assess *Pfpm2* gene copy number

Copy number of *P. falciparum plasmepsin 2* gene determination consisted of several stages. Initially, DNA was extracted from the blood spots on filter paper according to the Wooden method [11] and purified using Qiagen Kit. The DNA extract were then used as templates in the amplification process of the copy number gene target *Pfpm2* and *Pftub* genes using quantification of the real time polymerase chain reaction (RT-qPCR) and assay parameters according to the Witkowski method. The primers used for *Pfpm2* gene were 5'-TGGTGATGCAGAAGTTGGAG-3' and 5'-TGGGACCCATAAATTAGCAGA-3', while for *Pftubulin* these were 5'-TGATGTGCGCAAGTGATCC-3' and 5'-TCCTTTGTGGACATTCTTCCTC-3' [20]. Each control and samples were quantified in triplicates for *Pfpm2* and *Pftub*. The 3D7 strain were quantified in 6 replicates for *Pfpm2* and *Pftub.* Interpretation of results and run validation followed the Witkowski method. The 3D7 strain line was included in each run as standard control for one copy of *Pfpm2* gene in 6 replicates. *Pfpm2* copy number was calculated by the 2-ΔΔCt method [20] and the value was rounded up.

### Statistical methods

This study used the excel Kaplan–Meier analysis template provided by the WHO. The template calculated automatically data in the entry 1 and 2 files. The results are expressed as success and failure cumulative incidence, with 95% confidence intervals.

## Results

**Fig. 2:**
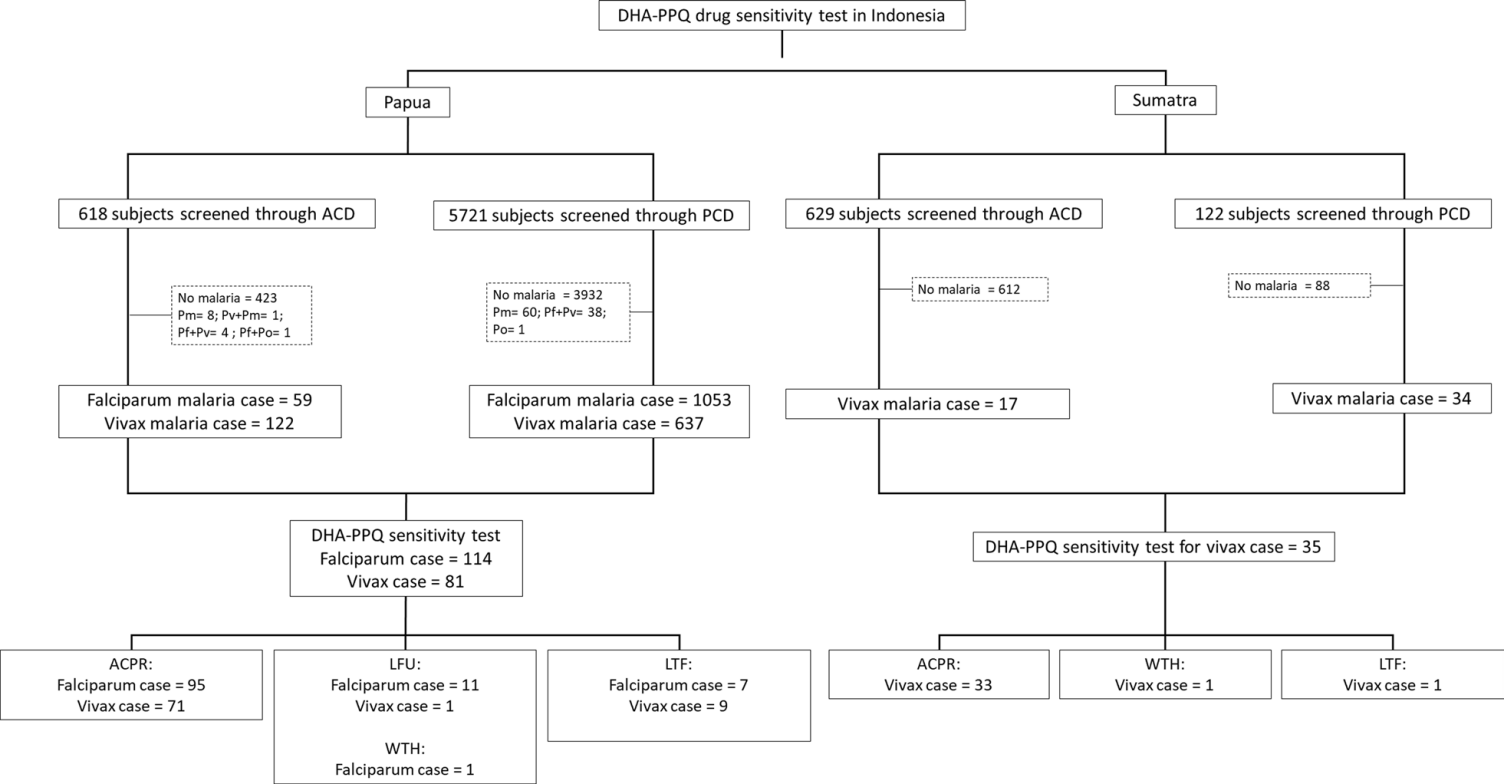
Flow chart sample collection in Papua and Sumatra

The subjects screening and recruitment in Papua and Jambi, Sumatra is shown in Fig. 2. Of the 6339 subjects screened through passive and active case detections in Papua, 1984 (31.3%) were found positive for malaria. Falciparum malaria dominated the malaria cases (56%) and followed by vivax malaria at 37.8%; 3.4% *Plasmodium malariae*; and 0.05% had *Plasmodium ovale*. The remainders were (2.1%) mixed infections.

### Demographic and parasitologic characteristics of the study populations

In Papua, a total of 114 (5.7%) of the 1984 *P. falciparum* cases and 81 (4.1%) *P. vivax* cases met inclusion criteria (Fig. 2). The remaining subjects were excluded due to age, inadequate asexual parasitaemia, unplanned travelling, refusal to provide consent, and local tribes war (unsecure situation for follow up activity).

The demographic characteristics of the enrolled study subjects in Papua and Sumatra has been shown in Table 1. In Papua, of the 114 enrolled subjects for falciparum cases, 56 were males and 58 were females with age ranging from 1 to 65 years (mean 22 years). The parasite density ranged from 600 to 213,000 per µl blood, whereas sexual stages (gametocytes) at day 0 were found in 5 subjects (4.4%) (Table 2). Of the 81 enrolled subjects for vivax cases, 41 were males and 40 were females with age ranging from 2 to 45 years (mean 15.1 years) (Table 1). At enrolment, the density of asexual forms ranged from 280 to 31,800 µl, whereas the sexual stages (gametocytes) at day 0 were found in 15 subjects (18.1%). At follow up, gametocytes were only found in 3 cases; 1 in day 2, 1 in day 3 and 1 in day 42 (Table 2).

**Table 1:** Baseline characteristics of study participants

Variable	Overall cases		
	Papua		Sumatra
	Falciparum	Vivax	Vivax
Number of persons enrolled (n)	114	81	35
Age (years)			
Mean (SD)	22 (16.8)	15.1 (12)	17 (14.6)
Range (y.o):	1–65	2–45	2–58
1–5	12	20	8
6–10	25	18	10
11–15	22	17	4
16–20	8	4	1
> 20	46	22	12
Gender			
Male [n (%)]	56 (49.1%)	41 (50.6%)	17 (48.6%)
Female [n (%)]	58 (50.9%)	40 (49.4%)	18 (51.4%)
Haemoglobin [g/dL]			
Mean (SD)	12.4 (1.8)	9.7 (3.2)	11.2 (7.3)
Range (min–max)	7.1–17.2	5.6–12.4	6.4–37.5
Body temperature [°C, mean (SD)]	37.6 (1.2)	37.4 (1.4)	36.8 (1.0)
Range	35–39.9	35–40.3	36–41
Parasite density (/µl) [mean geometric parasitaemia]	10,777	3918	1512
Range	600–213,000	280–31,800	320–9320

**Table 2:** Gametocyte of *P. falciparum* and *P. vivax* appearance by microscopy during the 42 days of follow up

Study site	Malaria case	Total sample at D0	Total sample with gametocyte appearance during days of follow-up									
			D0	D1	D2	D3	D7	D14	D21	D28	D35	D42
Papua	*P. falciparum*	114	5 (4.4%)	–*	2 (1.7%)	2 (1.7%)	3 (2.6%)	2 (1.7%)	1 (0.9%)	1 (0.9%)	0	0
	*P. vivax*	81	15 (18.5%)	–	1 (1.2%)	1 (1.2%)	0	0	0	0	0	1 (1.2%)
Sumatra	*P. vivax*	35	18 (51.4%)	–	0	0	0	0	0	0	1 (2.8%)	0

*No blood samples; D: Day

In Sumatra, 751 subjects screened through passive and active case detection and 51 (6.7%) were found positive for vivax malaria (Fig. 2). Of the 35 subjects (68.6%) that met the inclusion criteria and enrolled, 17 were males and 18 were females with age ranging from 2 to 58 years (mean 17 years). The parasite density ranged from 320 to 9320 µl (Table 1).

### Clinical and parasitological efficacy of DHA–PPQ for falciparum cases

Of the 114 falciparum cases enrolled, 102 cases completed the 42 day follow up, and 12 cases were either lost to follow up or withdrawn. Recurrent parasites of *P. falciparum* were detected in 7 cases at days 21, 35, day 42. Classification of the treatment outcomes by microscopy is presented in Table 3. At day 42, ACPR was noted in 93.1% (95% CI 86.4–97.2). Of the 7 LTF cases, one case was re-infected with *P. vivax*, 2 cases were confirmed as recrudescent infection and the remaining 4 cases were re-infection. Therefore, the PCR-corrected DHA–PPQ efficacy for falciparum was 97.9% (95% CI 92.7–99.7). No delay in parasite clearance nor severe adverse reaction was observed in any study participants.

**Table 3:** Treatment outcome from Papua and Sumatra Islands during the 42 days of follow up

Classification of treatment outcome			Papua				Sumatra	
			*P. falciparum*		*P. vivax*		*P. vivax*	
			n	% (95% CI)^#^	n	% (95% CI)	n	% (95% CI)
Total patient’s treatment failure and completed the 42 day follow up	Treatment Failure	ETF	0	–	0	–	0	–
		LCF	0	–	0	–	0	–
		LPF	7	6.9% (2.8–13.6)	9	11.3% (5.3–20.3)	1	2.9% (0.1–15.3)
	ACPR		95	93.1% (86.4–97.2)	71	88.8% (79.7–94.7)	33	97.1% (84.7–99.9)
Patients LFU/WTH			12	–	1	–	1	–
Total patients at baseline			114	–	81	–	35	–

ETF: Early treatment failure; LCP: Late clinical failure; LPF: Late parasitological failure; LFU: Lost to follow up; WTH: Withdrawn; ACPR: Adequate clinical and parasitological response

^#^Kaplan–Meier analysis

### Determination of the existence of SNPs in PfK13

PCR amplification and DNA sequencing of the *Pfk13* gene to observe the 20 SNPs associated with artemisinin resistance revealed that all *P. falciparum* isolates carried the wildtype allele.

### Pfpm2 gene copy number

Late treatment failure (LTF) was observed in 7 study participants with microscopy reading (Table 3) and genotyped for 6 samples using *msp1*, *msp2*, and *glurp* genes (Table 4). The delta Ct from 6 LTF were compare with Ct from control, *P. falciparum* strain 3D7. The estimation of the copy number from 6 LTF was calculated (Table 5). Three LTF (PAF 01, 08, and 19) had the same copy number of plasmepsin 2 in day 0 and day recurrence. Two LTF (PAF 37 and 112) have increased 2 copy number of plasmepsin 2 in day 0 and day recurrence, while 1 LTF (PAF 133) had 3 copy number. Of the 6 recurrent *P. falciparum* found, 2 indicated recrudescent and 4 cases were re-infection (Table 4). Analysis of the *Pfpm2* gene at day 0 and day of recurrence in recrudescent cases (PAF 01 and 19) revealed the same single copy number, whereas 3 of the 4 re-infection cases carried 2–3 copy numbers (Table 5).

**Table 4:** Genotyping results of the parasites at day 0 and day recurrence in *P. falciparum* cases

Isolate code	D0 Strain MSP1/MSP2/GLURP	DR Strain MSP1/MSP2/GLURP	Day of recurrence	Recrudescent/reinfection
PAF 01	K1^#^/FC27^+^/Code1*	K1/FC27/Code1	D21	Recrudescent/reinfection
PAF 08	K1/FC27/Code2	K1-RO33/FC27/Code3	D42	Reinfection
PAF 19	K1/FC27/Code1	K1/FC27/Code1	D35	Recrudescent/reinfection
PAF 37	K1/FC27/Code2	K1-RO33/FC27/Code3	D42	Reinfection
PAF 112	K1/FC27/Code3	MAD20/3D7/Code3	D42	Reinfection
PAF 133	K1/MAD20/FC27Code2	RO33/3D7/Code3	D35	Reinfection

^#^MSP1 amplicon: K1 = 150–300 base pairs (bp); MAD20 = 150–400 bp; and RO33 = 120–230 bp

^+^MSP2 amplicon: FC27 = 250–700 bp; 3D7 = 280–780 bp

*GLURP amplicon: Code1 = 501–600 bp; Code2 = 601–700 bp; and Code3 = 701–800 bp

**Table 5:** *Pfpm2* gene copy number of the parasites at day 0 and day recurrences in *P. falciparum* cases

Isolate number	PfPM2 copy number		Day of recurrence
	D0	DR strain	
PAF 01	1	1	D21
PAF 08	1	1	D42
PAF 19	1	1	D35
PAF 37	1	2	D42
PAF 112	1	2	D42
PAF 133	1	3	D35

### Clinical and parasitological efficacy of DHA–PPQ for vivax cases in Papua

Of the 81 vivax cases enrolled, 80 cases completed the 42 day follow up, and 1 case were lost to follow up. Classification of the treatment outcomes by microscopy was presented in Table 3 and showed 88.8% by microscopy (95% CI 79.7–94.7). LTF was observed in 9 study participants (11.2%) by microscopy. No delay in parasite clearance nor severe adverse reaction was observed.

### Clinical and parasitological efficacy of DHP for vivax cases in Sumatra

Of the 35 vivax cases enrolled, 34 cases completed the 42 day follow up, and 1 case were lost to follow up. Classification of the treatment outcomes by microscopy for vivax cases in Sumatra was presented in Table 3 and showed the efficacy of DHA–PPQ was 97.1% (95% CI 84.7–99.9%). One case (2.9%) showed recurrent infection at day 42 and categorized as LTF. No gametocyte was found after the treatment completed and during the follow up period up to day 42 except for 1 case where gametocyte appeared at day 35 (Table 2). Of the 34 vivax cases enrolled, no delayed parasite clearance was observed at Day 3.

### Gametocyte carriage during the treatment

Gametocytes was present at enrolment in 5 persons infected with *P. falciparum* and 15 persons with *P. vivax* malaria in Papua while in Sumatra 18 persons carried gametocyte at enrollment (Table 2). The proportion of persons with patent gametocytaemia in those with *P. falciparum* infection was 4.4% at D0, 1.7% at D2, 1.7% at D3, and 2.6% at D7. In Papua persons with *P. vivax* malaria, the proportion with gametocyte fell from 18.1% at D0 into1.2% at D2, D3 and D42, while in Sumatra the proportion of persons with patent gametocytaemia in those with *P. vivax* infection was 43.9% at D0 and 2.4% at D35 (Table 2).

## Glucose-6-phosphate dehydrogenase deficiency laboratory test

Of the 81 vivax cases enrolled in Papua and 35 vivax cases in Sumatra, the G6PD test using CareStart™ indicated a normal range as shown in the appearance of purple color in the cassette kit. All vivax cases in Papua and Sumatra were given primaquine with dose 0.25 mg/kg BW daily for a 14-days, starting at day 42 or day of recurrence. Primaquine was given to falciparum cases with single dose of 0.25 mg/kg BW at day 42 or day of recurrence.

## Discussion

Development and spread of the parasite resistance to the currently available artemisinin-based combination therapy (ACT) poses a substantial threat to the currently endorsed malaria elimination programme as it may increase not only malaria morbidity but also re-introduction of malaria in areas where elimination have been achieved. Results of this study clearly indicate that DHA–PPQ is still highly effective in both study sites, Papua and Sumatra. For the artemisinin particularly, this study showed no delay in parasite clearance and this is also supported by the absence of mutations associated with artemisinin resistance in *Pfk13* gene in any of the samples examined. As study in Papua New Guinea revealed the existence of *P. falciparum* isolates that carry the *Pfk13* gene C580Y mutation [19, 21], regular monitoring of the DHA–PPQ efficacy along the terrestrial border with PNG is mandatory to mitigate the spread of the artemisinin resistance to Indonesian Papua.

Evidence for the existence of parasite isolates that are slightly resistant to PPQ in Papua also alerts to the proper deployment of the drug in the area [22, 23]. Piperaquine resistance is associated with the increased copy number of the *Pfpm2* gene [20], and as a result the treatment failed to completely eliminate the parasite from the blood or prevent reinfection during the follow up period. The finding on the presence of 2 recrudescent cases at days 21 and 35 and re-infection at days 35–42 in this study may indicate the presence of *Plasmodium falciparum* isolates that survived the PPQ treatment. Analysis of the *Pfpm2* gene also supports for the existence of *P. falciparum* isolates that carried more than one copy of *Pfpm2* gene among the samples in Papua. Previous therapeutic efficacy study to evaluate the efficacy of DHA–PPQ in Southern part of Papua (Mimika Regency) did not find any *P. falciparum* isolates that carry more than one copy number in *Pfpm2* gene [22]. In Cambodia, amplification of the *plasmepsin* 2–3 gene cluster has been identified as an important molecular determinant of piperaquine resistance in *P. falciparum* [24–27] and resistance to piperaquine in fact increase the sensitivity of the mefloquine [24].

The finding for DHA–PPQ late treatment failure in this study alerts to the proper treatment of malaria in the area and also anticipate having second-line ACT to replace the piperaquine as partner drug. Currently, the Indonesia national policy to use quinine as second line drug is regarded to be impractical as it introduces longer treatment period and also more often side effects. In this regard, consideration of using another artemisinin-based combination, such as Artemether + lumefantrine or Artesunate + mefloquine at least for falciparum cases might be rational. Overall, the results of this study are reassuring and suggest that in the absence of artemisinin resistance, the artemisinin regimen in any artemisinin-based combination may delay de novo emergence of resistance to the partner drug, such as DHA–PPQ, artemether–lumefantrine and artesunate–mefloquine. DHA–PPQ has been used in Indonesia as the first line anti-malarial drug since 2008 [8, 10] and it took almost 10 years to first detect the early sign of resistance to the partner drug, piperaquine.

The high gametocyte carriage at enrolment in vivax cases (18.5% and 51.4% in Papua and Sumatra respectively) (Table 2) is likely associated with the poor accessibility to treatment and also compliance to the treatment regimen. Almost 50% of the subjects had received previous DHA–PPQ but never completed the 14-day primaquine treatment as recommended. To promote the safety prescription of 14-day primaquine policy, access to the G6PDd test in areas where vivax malaria still exists need to be prioritized. Currently, several G6PD deficiency test kit that met the criteria as point-of care (PoC) test at primary health centre could support the programme [28]. Incomplete treatment of vivax cases, particularly with primaquine requires special attention as it may expedite the emergence of parasite resistance to ACT as well as support for the local transmission.

In Indonesia, DHA–PPQ procurement is highly regulated by the Indonesian Ministry of Health, and the drug is only available at government health facilities and selected private-sector facilities, which are able to confirm that the prescription should be based on malaria positivity by microscopy or rapid diagnostic test. With this tight regulation it is anticipated that DHA–PPQ will continue to play a role in the treatment of uncomplicated malaria in Indonesia until malaria is successfully eliminated in the country. On the other aspect, implementation of evidence-based vector control may also contribute to mitigate transmission and delay the emergence of anti-malarial drug resistance.

## Conclusions

The therapeutic efficacy study conducted in two sentinel sites in Papua and Sumatra, Indonesia during 2017–2018 revealed that DHA–PPQ is still highly effective in both sites. The appearance of recurrent falciparum infection in small number of cases in Papua alert to the possible emergence of piperaquine resistance in the area and deserve further investigation to contain its spread and anticipate for the rational option of a second-line ACT. Further studies are required in different regencies in Papua, particularly those in border area with Papua New Guinea to determine the spread of resistance to DHA–PPQ.

## Data Availability

All relevant data are within the manuscript.
